# Circ-104792/miR-133a/Bcl-xL influences the proliferation and function of human trophoblastic and decidual stromal cells involved in recurrent abortion disease

**DOI:** 10.3389/fgene.2026.1707900

**Published:** 2026-02-12

**Authors:** Wenjuan Ma, Yuan Ma, Hongli Zhu, Panpan Shi, Xiaochun Huang, Yang Yang

**Affiliations:** 1 Department of Obstetrics and Gynecology, Xi’an People’s Hospital (Xi’an Fourth Hospital), Xi’an, Shaanxi, China; 2 Department of Obstetrics and Gynecology, Reproductive Medicine Center, Tangdu Hospital, Air Force Military Medical University, Xi’an, Shaanxi, China; 3 Reproductive Medicine Center, Xi’an People’s Hospital (Xi’an Fourth Hospital), Xi’an, Shaanxi, China

**Keywords:** Bcl-XL, cell proliferation, circ-104792, miR-133a, recurrent spontaneous abortion

## Abstract

**Background:**

The circRNA-miRNA axis is critically implicated in the pathogenesis of recurrent spontaneous abortion (RSA) by modulating trophoblast and decidual stromal cell functions. This study investigates the role of the miR-133a/circ-104792 network in RSA.

**Methods:**

Using qRT-PCR, we measured miR-133a and circ-104792 expression in chorion and decidua from 10 RSA patients and 10 controls. The functional effects on proliferation (CCK-8, EdU) and apoptosis (flow cytometry, TUNEL) were assessed in HTR-8/SVneo and HESC cells following transfection with miR-133a mimics/inhibitor or circ-104792. Direct targeting of Bcl-xL by miR-133a and its interaction with circ-104792 were validated via dual-luciferase reporter and RNA pull-down assays. Bcl-xL expression was evaluated by qRT-PCR and western blot.

**Results:**

miR-133a expression was (in chorion: p = 0.0008, 95% CI [2.85, 6.41]; in decidua: p = 0.0009, 95% CI [2.67, 6.03]) increased, while circ-104792 was significantly downregulated (in chorion: p = 0.0008, 95% CI [0.15, 0.34]; in decidua: p = 0.0007, 95% CI [0.13, 0.31]) in RSA tissues. *In vitro*, miR-133a overexpression inhibited cell proliferation, promoted apoptosis, and reduced Bcl-xL levels (in HTR-8/SVneo: 0.46-fold vs. 1.00 in NC group, p = 0.0045; in HESCs: 0.49-fold vs. 1.00 in NC group, p = 0.0003). Conversely, circ-104792 overexpression enhanced proliferation, suppressed apoptosis, and increased Bcl-xL expression (in HTR-8/SVneo: 2.17-fold vs. 1.00 in vector group, p = 0.0018; in HESCs: 1.94-fold vs. 1.00 in vector group, p = 0.0015). The dual-luciferase assay confirmed Bcl-xL as a direct target of miR-133a, and the RNA pull-down confirmed the miR-133a/circ-104792 interaction. Critically, circ-104792 overexpression rescued the suppressive effects of miR-133a on proliferation and Bcl-xL expression.

**Conclusion:**

Our findings demonstrate that miR-133a promotes RSA-associated cellular dysfunction by targeting Bcl-xL, while circ-104792 acts as a ceRNA to sponge miR-133a, thereby antagonizing its effects. The miR-133a/circ-104792/Bcl-xL axis represents a potential key regulatory network in RSA, presenting potential novel targets for diagnosis and therapy.

## 1 Introduction

Recurrent spontaneous abortion (RSA) is characterized by the loss of two or more pregnancies in succession, all taking place before the 20th week of gestation. This condition affects around 5% of women in their reproductive years. Etiologically, heredity, endocrine, infection, and immune parameters are the main pathogenic factors of RSA. However, the underlying causes of approximately 50% of RSA cases remain unidentified (La et al., 2021; Practice Committee of the American Society for Reproductive Medicine, 2006), presenting a significant challenge to understanding, preventing, and treating the condition.

Non-coding RNAs, such as lncRNAs, miRNAs, and circRNAs, are crucial in overseeing transcription and protein synthesis. MiRNAs, which are small RNA molecules about 20–24 nucleotides in length, primarily modulate gene expression after transcription by hindering translation or breaking down target mRNAs. These highly conserved molecules have become a major focus of research, with over 1,000 miRNAs identified as regulators of various biological activities, including cell proliferation, differentiation, and immune system regulation, are influenced by various factors. Some of these miRNAs are also suspected to contribute to pregnancy-related complications (Vishnoi and Rani, 2017; Morales-Prieto et al., 2013; Karin-Kujundzic et al., 2019). Recently, accumulating evidence indicates that the abnormal expression profile of miRNA in decidua or villi participates in the pathogenesis of RSA by regulating the growth and multiplication of cells, apoptosis, the infiltration and movement of trophoblast cells, along with the formation of blood vessels and the growth of the placenta (Wu et al., 2022).

CircRNAs known for their unique closed loop and single-stranded structure function as miRNA sponges due to their multiple miRNA binding sites, which interact with miRNAs and relieve miRNAs-induced inhibition of target genes. Research has shown that circRNAs contribute to the development of RSA by influencing the circRNAs-miRNAs-mRNAs signaling pathway (Rong et al., 2017; Liu J. et al., 2022). Previously, miRNA-133a was believed to be a muscle specific miRNA which participate in the regulation of muscle development (Miretti et al., 2017; Yao et al., 2022). Meanwhile, miRNA-133a downregulation has been reported in certain human malignances (Ji et al., 2013). Furthermore, extracellular vesicles, particularly those containing circRNAs and miRNAs, may also serve aspotential biomarkers of pathological pregnancy and could offer advantages in disease diagnosis as they enable non-invasive testing (Karin-Kujundzic et al., 2019).

Bcl-xL, produced by the BCL2L1 gene on chromosome 20 at band q11.21, has two isoforms resulted from alternative splicing. The shorter isoform, Bcl-xS, is made up of three exons, while the longer one, Bcl-xL, contains four exons. Inhibition of Bcl-xL can switch the p53 transcriptional response from senescence to apoptosis in cancer cells (Lucianò et al., 2021). MiRNAs contribute to RSA by directly suppressing anti-apoptotic genes like Bcl-xL and impairing trophoblast cell survival, invasion, or immune tolerance at the maternal-fetal interface. CircRNAs act as competing endogenous RNAs (ceRNAs) to sponge RSA-related miRNAs, thereby alleviating their repression of protective targets like Bcl-xL and promoting trophoblast function. Bcl-xL, as a key anti-apoptotic protein, counteracts mitochondrial apoptosis in trophoblasts and decidual cells, with its dysregulation (via miRNA/circRNA interactions) linked to placental insufficiency in RSA. The miRNA-circRNA-Bcl-xL axis forms a critical regulatory network in RSA, where circRNA downregulation or miRNA overexpression disrupts Bcl-xL-mediated cell survival, driving recurrent pregnancy loss.

Interestingly, findings regarding the role of miRNA-133a in RSA have been complex and context-dependent. While some earlier clinical observations, including from our group, reported a downregulation pattern, the present study was designed to specifically investigate its functional role in a defined cellular context. Contrary to the initial clinical observation, our current and more focused analysis revealed a significant upregulation of miR-133a in the chorion and decidua tissues of RSA patients. However, there remains a paucity of evidence on the relationship between miRNA-133a and RSA, and whether miRNA-133a participates in occurrence and progression of RSA via miRNA-133a/Bcl-xL remains still unknown. In this work, we provided a comprehensive understanding of how miR-133a and circ-104792 regulate Bcl-xL expression in RSA and highlight new candidate markers worthy of further investigation for the early identification and therapeutic intervention of RSA.

## 2 Materials and methods

### 2.1 Patients and samples

From January to December 2021, the obstetrics and gynecology department at Xi’an People’s Hospital (Xi’an NO.4 Hospital) was the site of the study. Participants based on uniform inclusion and exclusion criteria were applied, and data regarding reproductive history, such as menopausal status, parity and abortion history, were collected. Collected tissue samples including chorion and decidua tissues from 10 RSA patients and 10 women with normal pregnancies collected from routine prenatal diagnosis or during delivery were immediately sliced, treated with TRIzol reagent (Invitrogen, 15596018CN), and stored at −80 °C until further analysis. The study was authorized by the Ethics Committee of Xi’an People’s Hospital for Biomedical and Pharmaceutical Technologies (NO.20220032), with all participants provided written informed consent prior to their inclusion in the study.

### 2.2 Cell culture and transfection

The human trophoblast cell line HTR-8/SVneo was obtained from the obstetrics and gynecology department at Air Force Medical University Tangdu Hospital. Primary human endometrial stromal cells (HESCs) were isolated from endometrial tissues obtained from healthy women undergoing benign gynecological surgery at the same institution. The use of these tissues was approved by the relevant ethics committee, and informed consent was obtained from all donors.

HTR-8/SVneo cells were maintained in DMEM/F12 medium containing 10% FBS, 100 IU/ml penicillin, and 10 mg/ml streptomycin. HESCs were cultured in DMEM/F12 medium supplemented with 10% charcoal-stripped FBS, 1% insulin-transferrin-selenium (ITS), 100 IU/ml penicillin, and 10 mg/ml streptomycin. All cells were incubated at 37 °C with 5% CO_2_.

The identity and functional capacity of the HESCs were confirmed by their *in vitro* decidualization response. As presented in the Results section (Figure 6), treatment with a decidualization stimulus (e.g., a combination of medroxyprogesterone acetate (MPA) and cAMP analogue, or as per your actual method) led to a marked increase in the secretion of classic decidualization markers, insulin-like growth factor-binding protein 1 (IGFBP-1) and prolactin (PRL), as detected by immunofluorescence staining.

MiR-133a mimics, miR-133a inhibitors, and circ-104792 overexpression vector along with their corresponding negative controls (NC mimics, NC inhibitor, empty vector) were sourced from GenePharma. All transfections were performed using Lipofectamine™ 2000 (Invitrogen, 11,668,030). After a 48 h incubation, transfected cells were harvested for subsequent analysis.

### 2.3 RNA isolation and quantitative real-time polymerase chain reaction (qRT-PCR)

Total RNA was extracted using the TRIzol reagent from Takara. For the qRT-PCR, we utilized the QuantStudio SYBR Green One-Step qRT-PCR Kit from ABI, following the guidelines set by the manufacturer. The qRT-PCR analysis took place on the ABI 7500 Fast PCR System, and utilized SYBR Green PCR Master Mix from Takara, with either β-actin or U6 serving as the internal controls. The levels of gene expression relative to controls were calculated using the 2^–ΔΔ Cycle threshold value (Ct)^ method (Livak and Schmittgen, 2001). Primers, synthesized by Tsingke, are listed below and See Supplementary Table S1: Hsa-miR-133a (Loop primer: GTC​GTA​TCC​AGT​GCA​GGG​TCC​GAG​GTA​TTC​GCA​CTG​GAT​ACG​ACC​AGC​TG, Forward: TGC​GCT​TTG​GTC​CCC​TTC​AAC​C, Reverse: CCA​GTG​CAG​GGT​CCG​AGG​TAT​T); U6 (Forward: CGC​TTC​GGC​AGC​ACA​TAT​AC, Reverse: AAA​TAT​GGA​ACG​CTT​CAC​GA); Homo-circ-104792 (Forward: AAG​AAG​TGG​CTG​TAG​GGA​GCA​TAG, Reverse: TGC​CTC​ACA​GAA​CAG​TCT​CCA​TAC); Homo BCL-XL (Forward: CTG​AAT​CGG​AGA​TGG​AGA​CC, Reverse: TGG​GAT​GTC​AGG​TCA​CTG​AA); Homo-β-actin (Forward: CCC​TGG​AGA​AGA​GCT​ACG​AG, Reverse: CGT​ACA​GGT​CTT​TGC​GGA​TG).

### 2.4 Immunoblotting

To perform immunoblotting, cells were lysed using cold RIPA buffer (servicebio, G2002) mixed with a protein inhibitor cocktail containing PMSF (servicebio, G2008) and phosphatase inhibitor (servicebio, G2007) to extract the proteins. A BCA kit (Gbs, G3522-3) was utilized to evaluate the protein concentrations of the lysate. Samples were separated using SDS-PAGE (12% separating gel and 5% stacking gel) and subsequently transferred onto polyvinylidene fluoride (PVDF) membranes (Millipore, IPVH00010) using a semi-dry transfer system (Nanjing EYEN, FW606) with S-TRANS anode buffer (ACE, AL011-01–1) and S-TRANS cathode buffer (ACE, AL011-01–2) at 1.5 A for 420 s. Following this, the membranes were treated with 5% non-fat milk in TBST (servicebio, G0004) for blocking at room temperature for 2 h and incubated overnight at 4 °C with the appropriate primary antibodies: rabbit polyclonal anti-Bcl-xL (Affinity, AF6414) at a dilution of 1:1,000 and mouse monoclonal anti-β-actin (Affinity, T0022) at a dilution of 1:5,000. On the next day, the membranes underwent washing with TBST buffer 5 times (5 min each) and then incubated with HRP-conjugated secondary antibodies-goat anti-rabbit IgG (Biyuntian, A0208) and goat anti-mouse IgG (Wuhan Sanying Biotechnology, SA00001-1)-at a dilution of 1:10,000 for 2 h at room temperature. After another 5 washes with TBST, the membranes were incubated with ECL substrate (servicebio, G2014). All Western blot images were acquired using X-ray film (FUJIFILM, 4,741,019,274) following enhanced chemiluminescence detection. Protein bands were visualized by exposing PVDF membranes to X-ray film under darkroom conditions, followed by development and fixation using a developing and fixing kit (Tianjin Hanzhong Photographic Materials Factory), consistent with standard protocols for chemiluminescent detection.

### 2.5 Apoptosis assay

To detect cell apoptosis, staining was performed using FITC-Annexin V and propidium iodide (PI) (KeyGen Biotech, KGA108). HTR-8/SVNEO and HESC were cultured in six-well plates for 24 h before subjected to transfection with miR-133a mimics and the miR-133a inhibitor, circ-104792 or the relevant negative control (NC mimics, NC inhibitor, vector). 48 h later, the cells underwent trypsinization and were subsequently rinsed with PBS. Next, thesuspension was adjusted to 10^6 cells/ml using the binding buffer. Finally, 5 µl FITC-Annexin V was added to cells and incubated in dark for 5 min, following with 5ul PI and incubation for 10 min. Cell samples were finally detected by a flow cytometry (BECKMAN, CytoFLEX).

### 2.6 Cell proliferation assay

Before transfection with plasmid DNA or negative controls, HTR-8/SVneo and HESC cells were seeded at a density of 5 × 10^3^ per well in either six-well or 96-well plates. Following a 48 h incubation, cell proliferation was assessed using an EdU assay kit (Abbkine, KTA 2030) or CCK8 (MCE, HY-K0301) according to the directions given by the manufacturer.

### 2.7 Dual-luciferase reporter assays

The Bcl-xL promoter was inserted into a Bcl-xL basic plasmid (Generay, Shanghai). To perform the luciferase assay, transfection was performed on HEK293T cells that were grown in 24-well plates using Lipofectamine™ 3,000 (Invitrogen, 11,668,019). The components of the transfection mixture included 20 ng plasmids encoding Renilla luciferase, 200 ng firefly luciferase reporter plasmids, and a range of combinations involving the empty psiCHECK™-2 vector, psiCHECK™-2-circ-104792, psiCHECK™-2-miR-133a mimics, or psiCHECK™ -2-circ-104792 and psiCHECK™-2-miR-133a mimics plasmids. The Dual-Lumi™ Luciferase Reporter Gene Assay Kit (Beyotime, RG027) was utilized to measure luciferase activity, in accordance with the manufacturer’s guideline (Luo et al., 2023).

### 2.8 RNA pull-down

Using the Magna RNA Binding Protein Immunoprecipitation Kit (Millipore, 17–700) as previously described (Chen et al., 2019), the RNA capture assay was performed. The supernatant was then incubated with 10 μg biotin-labeled Homo-circ-104792 and control probes provided by Tsingke. After an overnight incubation, the probe-complexes were mixed with Pierce™ Streptavidin Magnetic Beads (Thermo, 88,816) in washing buffer for 1 h at room temperature. After centrifuging the magnetic beads-probe complexes, they were washed for six times with washing buffer. The RNA immunoprecipitated was then analyzed using qRT-PCR.

### 2.9 Immunofluorescence

The process began with fixing cells with 4% paraformaldehyde for 15 min at room temperature (RT). Following this, 0.5% Triton-X-100 was used to permeabilize the cells for 10 min at RT, then cells were incubated in a 5% BSA solution in PBS for 1 h at ambient temperature for blocking. Main antibodies against IGFBP-1 (Abcam, 1:100) and PRL (Abcam, 1:100) were applied and incubated overnight at 4 °C followed by a 1-h incubation with a Cy3-conjugated secondary antibody (Boster, BA1032) at RT. Images were captured using a fluorescence microscope model BX53 (Olympus) after the nuclei were stained with 4,6-diamidino-2-phenylindole (DAPI) for 5 min at RT.

### 2.10 Statistical analysis

All data were analyzed in *GraphPad Prism* 10 software and presented as the mean values ± Standard Error Mean (mean ± SEM). The one-way ANOVA test or Student’s t-test was utilized to assess statistical significance. For examining statistical correlation, Pearson’s correlation coefficient was applied. Differences with *p* < 0.05 were considered statistically significant.

### 2.11 Sample size and power analysis

Prior to the study, a power analysis was performed using G*Power 3.1 software to determine the minimum sample size required to detect a meaningful effect. Based on preliminary data and previous literature reports on miRNA/circRNA expression differences in RSA (Qin et al., 2016; Wang et al., 2012), we assumed an effect size (Cohen’s d) of 1.2 (large effect), a significance level (α) of 0.05, and a statistical power (1-β) of 0.8. The analysis indicated that a sample size of 8 cases and 8 controls would be sufficient to detect the anticipated differences in miR-133a and circ-104792 expression. To account for potential technical variability and sample loss, we enrolled 10 RSA patients and 10 controls, which exceeded the minimum required sample size and ensured adequate power to detect the observed effects. For *in vitro* experiments, each group was performed in triplicate, and the sample size was determined based on similar *in vitro* studies investigating trophoblast and decidual cell function (Luo et al., 2023) ensuring that the experiments had sufficient power to detect significant differences in cell proliferation, apoptosis, and gene expression.

## 3 Results

### 3.1 miR-133a was upregulated in both chorion and decidua tissues from RSA patients

**FIGURE 1:**
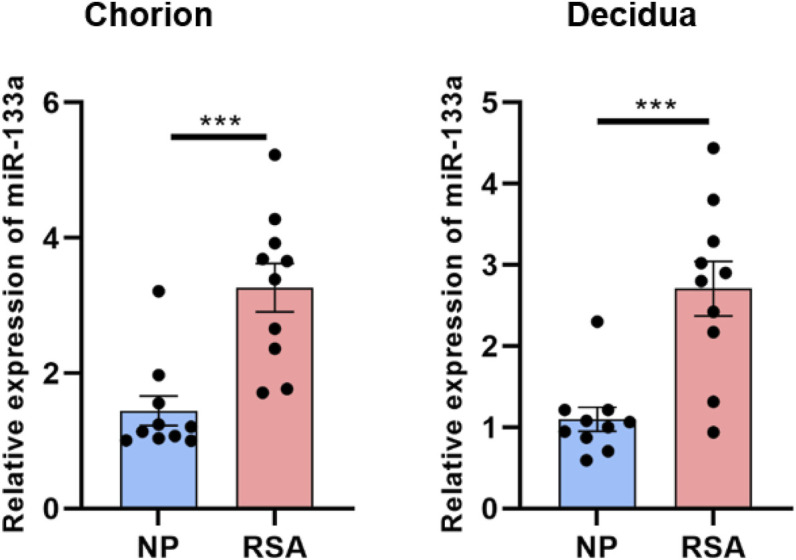
miR-133a was upregulated in patients with recurrent spontaneous abortion (RSA) Comparison of relative expression of miR-133a in chorion and decidua tissue were quantified by qRT-PCR in women with normal pregnancy or RSA. The results are shown as mean ± SEM. ***P < 0.001; SEM, standard error of the mean; RSA, recurrent spontaneous abortion.

To assess miR-133a expression levels, qRT-PCR was conducted on chorion and decidua tissues from 10 RSA patients and 10 women with normal pregnancies. The findings indicated a increase of miR-133a expression in the chorion (p = 0.0008, 95% CI [2.85, 6.41]) and decidua tissues (p = 0.0009, 95% CI [2.67, 6.03]) of RSA patients compared to those from women with normal pregnancies (Figure 1).

### 3.2 miR-133a inhibited cell proliferation and increased cell apoptosis of HTR-8/SVneo cells and HESC cells

**FIGURE 2:**
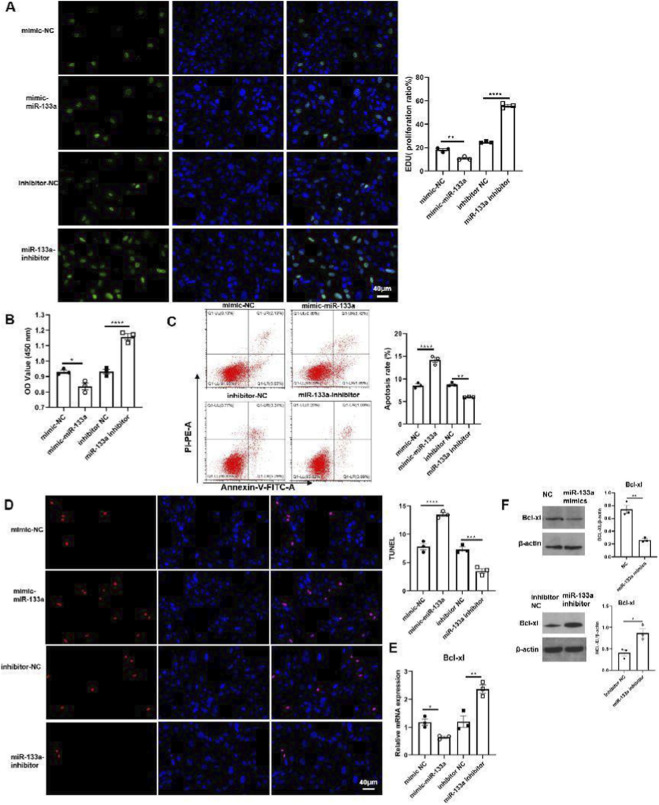
The apoptosis rate of HTR8 was increased by miR-133a while restrained by miR-133a inhibitor. **(A)** HTR-8/SVneo cells were transfected with mimics negative control (NC)/miR-133a mimics or inhibitor NC/miR133a, then treated with EdU for 6 h prior to click reaction, and the analysis was performed to quantify EdU-positive cells based on individual DAPI signal. **(B)** The cell viability of HTR-8/SVneo was examined and analyzed using CCK8. **(C,D)** The apoptosis ratio was analyzed by flow cytometry or by quantifying TUNEL-positive cells in HTR-8/SVneo cells transfected with mimics NC/miR-133a mimics or inhibitor NC/miR133a. **(E)** The mRNA expression of Bcl-xL in HTR8 cells transfected with mimics NC/miR-133a mimics or inhibitor NC/miR133a. **(F)** The protein level of Bcl-xL in HTR-8/SVneo cells transfected with mimics NC/miR-133a mimics, inhibitor NC/miR133a. *P < 0.05, **P < 0.01, ***P < 0.001, ****P < 0.0001.

**FIGURE 3:**
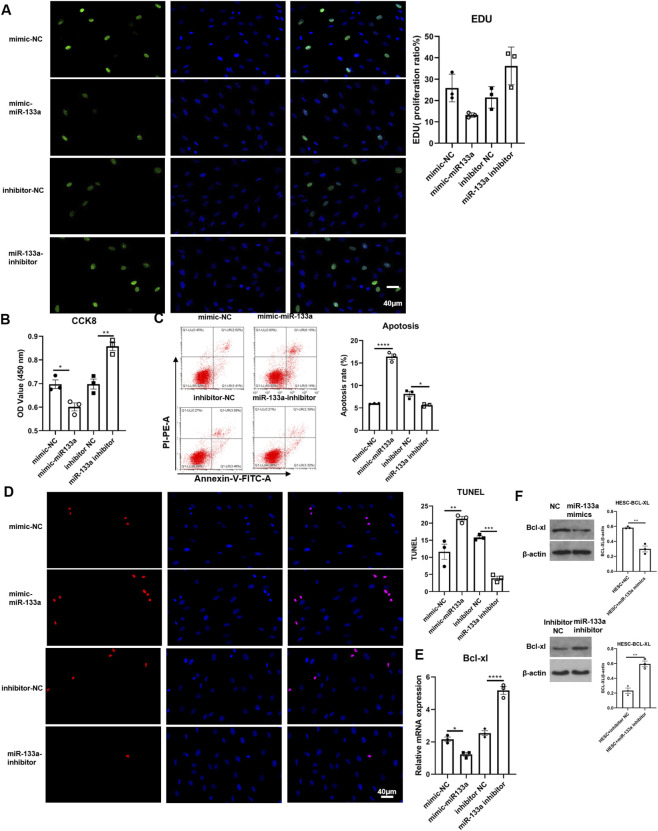
miR-133a was negatively correlated with the expression of Bcl-xL in HESC. **(A)** HESC cells were transfected with mimics NC/miR-133a mimics or inhibitor NC/miR133a. They were treated with EdU for 6 h prior to click reaction, and the analysis was performed to quantify EdU-positive cells based on individual DAPI signal. **(B)** The cell viability of HESC was examined and analyzed using CCK8. **(C,D)** The apoptosis ratio was analyzed by flow cytometry or was performed to quantify TUNEL-positive cells in HESC cells transfected with mimics NC/miR-133a mimics or inhibitor NC/miR133a. **(E)** The mRNA expression of Bcl-xL in HESC cells transfected with mimics NC/miR-133a mimics or inhibitor NC/miR133a. **(F)** The protein level of Bcl-xL in HESC cells transfected with mimics NC/miR-133a mimics, inhibitor NC/miR133a. *P < 0.05, **P < 0.01, ***P < 0.001, ****P < 0.0001.

To assess the impact of miR-133a on RSA, we transfected HTR-8/SVneo and HESC cells with either NC/miR-133a or miR-133a inhibitor. The EdU and CCK8 assays revealed a significant decrease in cell proliferation in the miR-133a transfected cells compared to the NC group (CCK8: p = 0.0018, 95% CI [0.21, 0.45]), while this effect was counteracted by the miR-133a inhibitor (Figures 2A,B; Figures 3A,B). As further detected through flow cytometry and TUNEL assay, miR-133a increased the cell apoptosis (p = 0.0009, 95% CI [3.25, 7.68]), while reduced it when inhibited (Figures 2C,D; Figures 3C,D). Bcl-xL expression was assessed through qRT-PCR and Western blot methods. Given that Bcl-xL inhibition is known to promote cell apoptosis by activating p53 (Bharti et al., 2022), our results indicated that Bcl-xL levels significantly decreased upon miR-133a overexpression (in HTR-8/SVneo: 0.46-fold vs. 1.00 in NC mimics group, p = 0.0045; in HESCs: 0.49-fold vs. 1.00 in NC mimics group, p = 0.0003), Conversely, inhibiting miR-133a led to a notable increase in Bcl-xL levels in both HTR-8/SVneo (2.09-fold vs. 1.00 in inhibitor NC group, p = 0.0027) and HESCs (2.04-fold vs. 1.00 in inhibitor NC group, p = 0.0001) (Figures 2E,F; Figures 3E,F). Collectively, these observations suggest that miR-133a may have a detrimental effect on HTR-8/SVneo cells and HESCs.

### 3.3 circ-104792 promoted cell proliferation in HTR-8/SVneo cells and HESC cells

**FIGURE 4:**
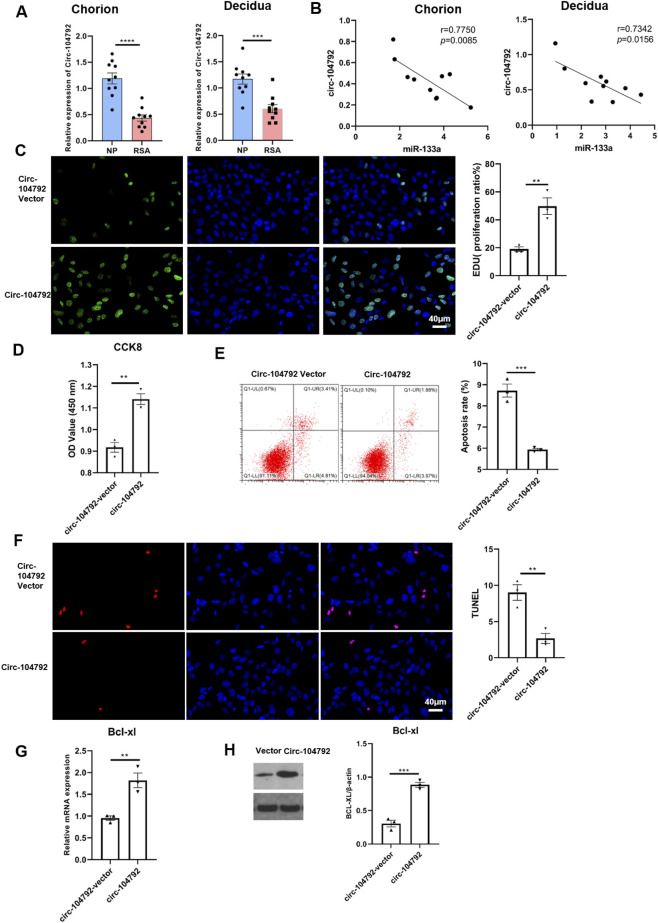
The cell apoptosis was decreased by circ-104792 both in HTR8/Snevo cells. **(A)** Comparison of relative expression of circ-104792 in chorion and decidua tissue were quantified by qRT-PCR in women with normal pregnancy or RSA. **(B)** Pearson’s correlation analysis was used to analyze the association between miR-133a and circ-104792 expressions in chorion and decidua from RSA. **(C)** HTR-8/SVneo cells were transfected with circ-104792 vector or circ-104792. And the analysis was performed to quantify EdU-positive cells based on individual DAPI signal. **(D)** The cell viability of HTR-8/SVneo cells was examined and analyzed using CCK8. **(E,F)** The apoptosis ratio was analyzed by flow cytometry or TUNEL intensities in HTR-8/SVneo cells transfected with circ-104792 vector/circ-104792. **(G)** The mRNA expression of Bcl-xL in HTR-8/SVneo cells transfected with circ-104792. vector/circ-104792. **(H)** The protein level of Bcl-xL in HTR-8/SVneo cells transfected with circ-104792 vector/circ-104792. **P < 0.01, ***P < 0.001, ****P < 0.0001.

**FIGURE 5:**
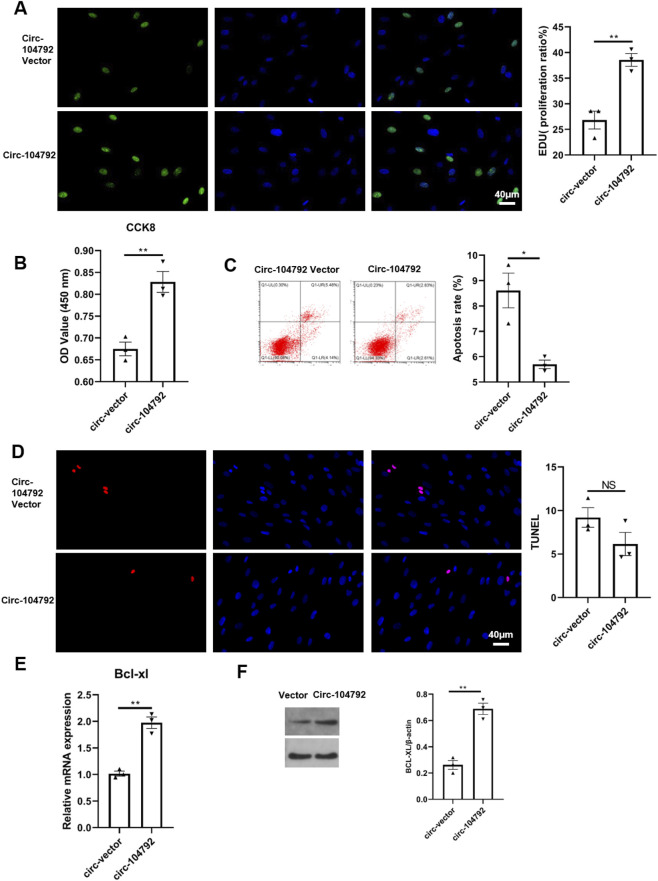
circ-104792 overexpression elevated cell proliferation of HESCs. **(A)** HESC cells were transfected with either circ-104792 vector or circ-104792. The analysis was performed to quantify the signal intensity in EdU-positive cells based on individual DAPI signal intensity. **(B)** The cell viability of HESCs was examined and analyzed using CCK8. **(C,D)** The apoptosis ratio was analyzed by flow cytometry or TUNEL intensities in HESCs transfected with circ-104792 vector/circ-104792. circ. **(E)**The mRNA expression of Bcl-xL in HESCs transfected with circ-104792 vector/circ-104792. **(F)** The protein level of Bcl-xL in HESCs cells transfected with circ-104792 vector/circ-104792. *P < 0.05, **P < 0.01.

Initially, the expression of circ-104792 was evaluated in chorion and decidua tissues from 10 RSA patients and 10 women with normal pregnancies through qRT-PCR. In comparison to the normal pregnancy group, the RSA group exhibited a reduction in circ-104792 levels in chorion (p = 0.0008, 95% CI [0.15, 0.34]) and decidua (p = 0.0007, 95% CI [0.13, 0.31]) (Figure 4A). Pearson’s correlation analysis further showed a negative correlation between miR-133a and circ-104792 expression in the chorion and decidua tissues of RSA patients (Figure 4B). To explore the function of circ-104792 in RSA, HTR-8 and HESC cells were subjected to transfection with vector/circ-104792. CCK8 assays revealed that cell proliferation was increased in circ-104792-overexpressing HTR-8/SVneo cells (p = 0.0019, 95% CI [0.25, 0.51]), with consistent results in HESCs (Figure 4D; Figure 5B).

In addition, flow cytometry and TUNEL assay also confirmed that circ-104792 expression repressed the apoptosis of HTR-8/SVneo cells (Figures 4E,F),which was also found in HESC cells (Figure 5C), although no obvious change was exhibited in the TUNEL assay (Figure 5D). Moreover, compared with the empty vector group, Bcl-xL mRNA and protein levels were elevated in HTR-8/SVneo (2.17-fold vs. 1.00, p = 0.0018) and HESCs (1.94-fold vs. 1.00, p = 0.0015) transfected with circ-104792 (Figures 4G,H; Figures 5E,F). In sum, circ-104792 may reduce apoptosis in HTR8 cells and HESC cells via Bcl-xL.

### 3.4 circ-104792 and miR-133a regulated IGFBP-1 and PRL expression in HESCs

To explore the functions of circ-104792 and miR-133a on the decidualization ability of HESCs. Key factors of decidualization, Insulin-like growth factor binding protein 1 (IGFBP-1) and prolactin (PRL) levels were detected through immunofluorescence assay. The results indicated that the levels of IGFBP-1 and PRL were elevated when transfected with circ-104792 compared to the vector group.

**FIGURE 6:**
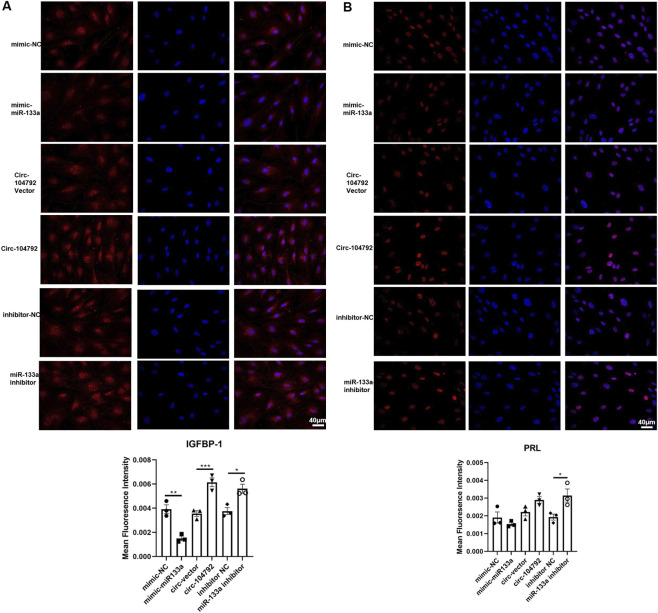
miR-133a and circ-104792 affected the expression of IGFBP-1 and PRL in HESCs. **(A)** The expression of IGFBP-1 was analyzed by immunofluorescence staining in HESC cells transfected with mimics NC/miR-133a mimics, inhibitor or circ-104792 vector/circ-104792 or inhibitor NC/miR133a. **(B)** The expression of PRL was analyzed by immunofluorescence staining in HESC cells transfected with mimicNC/miR-133a mimics, inhibitor or circ-104792 vector/circ-104792 or inhibitor NC/miR133a. *P < 0.05, **P < 0.01, ***P < 0.001.

Moreover, the expression of IGFBP-1 and PRL was reduced by miR-133a transfection group in comparison to the NC group (IGFBP-1: p = 0.0017, 95% CI [85.6, 320.8], PRL: p = 0.0028, 95% CI [120.5, 385.7]), while this was reversed when treated with miR-133a inhibitor (Figures 6A,B). Therefore, the results suggest that miR-133a induces dysfunction of HESCs through suppressing the expressions of IGFBP-1 and PRL and circ-104792 may play an opposite role.

### 3.5 circ-104792 positively regulated Bcl-xL expression via interaction with miR-133a

**FIGURE 7:**
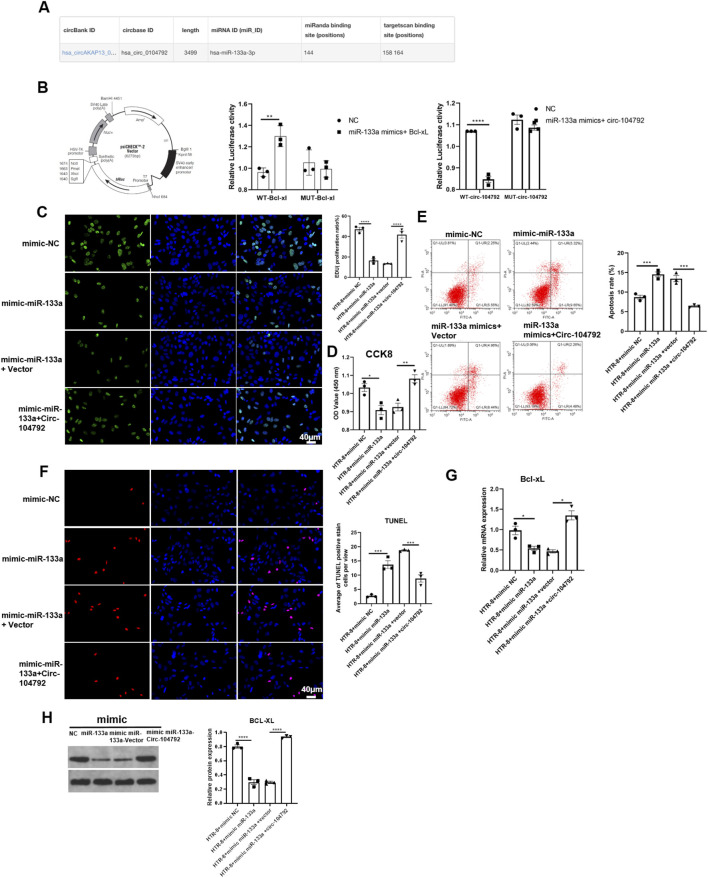
Bcl-xL was the target of miR-133a and circ-104792. **(A)** Bioinformatic prediction by *TargetScan* indicated that circ-104792 contains a putative miR-133a binding region. **(B)** A dual-luciferase reporter assay was used to confirm the combination between miR-133a and Bcl-xL and the interaction of miR-133a with circ-104792. A fragment of miR-133a that was predicted to bind to the Bcl-xL 3′-UTR was cloned into firefly luciferase psiCHECK^TM^-2 vector. The Renilla luciferase plasmid was co-transfected for normalizing luciferase activity. **(C)** HTR-8/SVneo cells were treated with EdU for 6 h prior to click reaction, and the analysis was performed to quantify EdU-positive cells based on individual DAPI signal. **(D)** The cell viability of HTR-8/SVneo was examined and analyzed using CCK8. **(E,F)** The apoptosis ratio was analyzed by flow cytometry **(E)** or TUNEL **(F)** in HTR-8/Snevo cells transfected with transfected with mimics NC/miR-133a mimics, miR-133a mimics-inhibitor or miR-133a mimics-vector/miR-133a mimics-circ-104792 or inhibitor NC/miR133a. **(G,H)** The expression (mRNA and protein) of Bcl-xL in HTR-8/SVneo cells transfected with mimics NC/miR-133a mimics, circ-104792 vector/circ-104792 or NC/miR133a. *P < 0.05, **P < 0.01 vs. corresponding controls.

The *TargetScan* website identified a possible target site for miR-133a on circ-104792 (Figure 7A). A dual-luciferase reporter assay was performed to explore this interaction. The results showed that overexpressing miR-133a caused a notable decrease in luciferase activity for the Bcl-xL-WT reporter (p = 0.0009, 95% CI [0.32, 0.75]), whereas it had minimal effect on the Bcl-xL-MUT reporter. Additionally, when both miR-133a and circ-104792 were overexpressed, there was a decrease in luciferase activity for the circ-104792-WT reporter (p = 0.0011, 95% CI [0.29, 0.71]), but no change was observed in the circ-104792-MUT reporter (Figure 7B). In separate experiments, HTR-8/SVneo cells were transfected with either NC/miR-133a or vector/circ-104792. These findings indicated that cells exhibited decreased proliferation following the overexpression of miR-133a, while circ-104792 overexpression mitigated the apoptosis induced by miR-133a, as measured by Edu and CCK8 assays (Figures 7C,D).

The flow cytometry and TUNEL assay also confirmed that circ-104792 expression repressed the cell apoptosis of HTR-8/SVneo but the miR-133a overexpression increased the cell apoptosis of HTR-8/SVneo (Figures 7E,F). QRT-PCR and Western blot revealed that Bcl-xL expression was reduced in the miR-133a group compared to NC. However, in the mimic miR-133a + circ-104792 group, Bcl-xL levels were higher than in the mimic miR-133a + vector group (Figures 7G,H). These findings collectively suggest that miR-133a and circ-104792 significantly influence the regulation of cell proliferation and apoptosis in HTR-8/SVneo cells by modulating Bcl-xL levels.

### 3.6 Circ-104792 improved cell functions of HESC and rescued cell dysfunction aggravated by miR-133a

**FIGURE 8:**
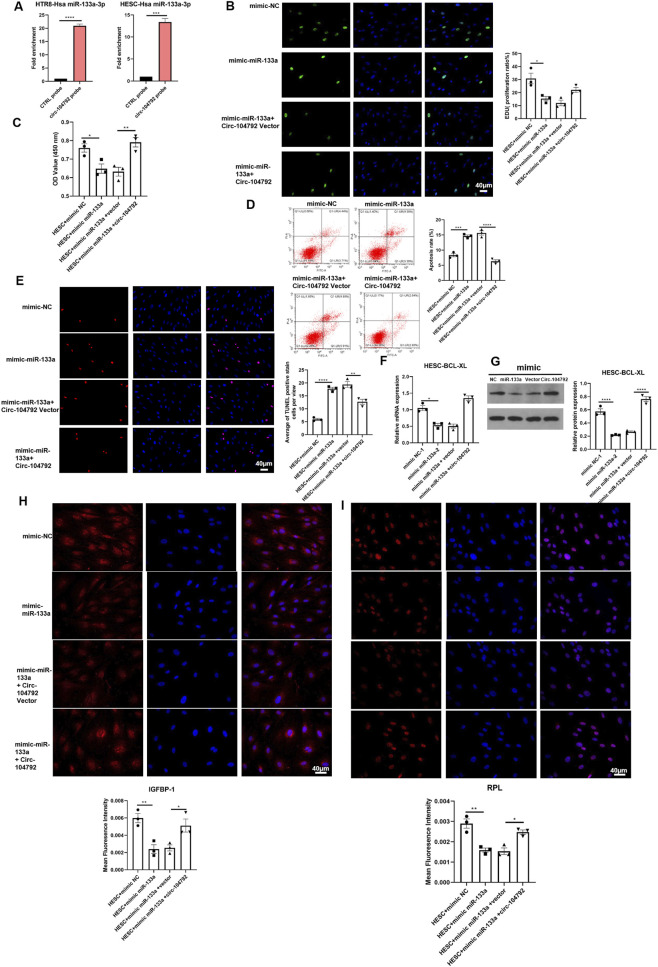
circ-104792 could improve cell functions of HESC and miR-133a aggravated cell dysfunction. **(A)** Enrichment efficiency of biotin tagged circ-104792 probes assessed by RNA pull-downs in HESC or HTR-8/SVneo cells. **(B)** HESC cells were overexpressed miR-133a before transfecting with mimic NC/miR-133a or vector/Circ-104792, and the analysis was performed to quantify EdU-positive cells based on individual DAPI signal. **(C)** The cell viability of HESC was examined and analyzed using CCK8. **(D,E)** The apoptosis ratio was analyzed by flow cytometry **(D)** or TUNEL **(E)** in HESC cells transfected with transfected with mimics NC/miR-133a mimics, miR-133a mimics -inhibitor or circ-104792 vector/miR-133a mimics -circ-104792 or inhibitor NC/miR133a. **(F)** The mRNA expression of Bcl-xL in HESC cells transfected with mimics NC/miR-133a mimics, miR-133a mimics -inhibitor or circ-104792 vector/miR-133a mimics-circ-104792 or inhibitor NC/miR133a. **(G)** The protein level of Bcl-xL in HESC cells transfected with mimics NC/miR-133a mimics, miR-133a mimics -inhibitor or circ-104792 vector/miR-133a mimics-circ-104792 or inhibitor NC/miR133a. **(H,I)** The expression of IGFBP-1 **(H)** or PRL **(I)** was analyzed by immunofluorescence staining in HESC cells transfected with mimics NC/miR-133a mimics, inhibitor or circ-104792 vector/circ-104792 or inhibitor NC/miR133a. *P < 0.05, **P < 0.01, ***P < 0.001, ****P < 0.0001.

Biotin-labeled circ-104792 was combined with total RNA, and magnetic bead-labeled streptavidin was used to capture the biotin-labeled complexes. The captured RNA was then analyzed through qPCR. The findings revealed that miR-133a-3p was more abundant in the circ-104792 probe group HTR8 cells (p = 0.0007, 95% CI [3.85, 9.26]) (Figure 8A), with consistent results in HESCs. Additionally, CCK-8 and EdU assays performed on HESCs showed that stable transfection with miR-133a mimics reduced cell proliferation. However, the proliferation inhibitory effect was reversed when circ-104792 was co-transfected with miR-133a mimics, as evidenced by the CCK-8 assay results (Figures 8B,C).

Furthermore, we performed flow cytometry and TUNEL assay along with transfections of mimic miR-133a accelerated cell apoptosis which can be reduced by transfecting mimic miR-133a + circ-104792 in HESCs (Figures 8D,E). In addition, the mRNA and protein levels of Bcl-xL were both found reduced in the miR-133a transfection group (Figures 8F,G), whereas transfections of mimic miR-133a + circ-104792 rescued the inhibition of Bcl-xL assessed by western blot (Figure 8G). Moreover, the expression level of IGFBP-1 and PRL was markedly decreased in mimic miR-133a transfected cells and enhanced in mimic miR-133a-circ-104792 transfected cells assessed by immunofluorescence staining (Figures 8H,I). These results suggest that miR-133a/circ-104792 may regulate proliferation and apoptosis via Bcl-xL in HESCs.

## 4 Discussion

RSA is a complex reproductive disorder which represents a serious risk to women’s health during their child-bearing years globally and there is a pressing need for effective diagnostic biomarkers. In recent year, Qin et al. discovered miRNA-320b, miRNA-146b-5p and miRNA-221–3p were upregulated and miRNA-101–3p was inhibited in RSA (Qin et al., 2016). In addition, miR-526b-5p had an effect on the proliferation, migration, and invasion of trophblasts by regulating c-Myc and Foxp1 in the development of RSA (Luo et al., 2023).

In the villi affected by RSA, there was a significant upregulation of miR-133a, which contributes to RSA development through the regulation of HLA-G expression (Wang et al., 2012). The findings indicated that miR-133a levels were higher (chorion: p = 0.0008, 95% CI [2.85, 6.41]; decidua: p = 0.0009, 95% CI [2.67, 6.03]), while circ-104792 levels were lower (chorion: p = 0.0008, 95% CI [0.15, 0.34]; decidua: p = 0.0007, 95% CI [0.13, 0.31]) in the chorion and decidua tissues of RSA patients, in comparison to tissues from normal pregnancies. This finding points to a potential crucial function of miR-133a in the pathogenesis of RSA. Moreover, circRNAs, which are endogenous non-coding RNAs, serve as valuable noninvasive biomarkers for RSA diagnosis. These circRNAs are involved in trophoblast proliferation, migration, and invasion, and disturbances in their expression can lead to placental dysfunction related to RSA (Li Z. et al., 2020). Additionally, we found that miR-133a was notably more abundant in the chorion (p = 0.0008, 95% CI [2.85, 6.41]) and decidua (p = 0.0009, 95% CI [2.67, 6.03]) tissues of 10 RSA patients than in those from women experiencing normal pregnancies. In addition, miR-133a inhibited cell proliferation, increased cell apoptosis and downregulated Bcl-xL expression of HTR-8/SVneo cells (0.46-fold vs. 1.00 in NC group, p = 0.0045)) and HESC cells (0.49-fold vs. 1.00 in NC group, p = 0.0003) *in vitro*. However, the expression of circ-104792 showed significantly lower both in chorion (p = 0.0008, 95% CI [0.15, 0.34]) and decidua (p = 0.0007, 95% CI [0.13, 0.31]) tissues samples with RSA, and circ-104792 overexpression in HTR-8/SVneo cells and HESC cells could promote cell proliferation, decrease cell apoptosisapoptosis and increase the expression of Bcl-xL 2.17-fold vs. 1.00 in vector group, p = 0.0018; in HESCs: 1.94-fold vs. 1.00 in vector group, p = 0.0015). Liu K et al. found that circ-NCX1 showed as a key regulator of LPS-induced chondrocyte apoptosis via the miR-133a/Sirt1 axis in the development of osteoarthritis (OA) (Liu K. et al., 2022). Furthermore, A body of research has identified that circRNA is crucial in regulating cell proliferation, apoptosis through regulating miRNAs engaged in numerous physiological and pathological functions (Yang J. et al., 2020; Li H. et al., 2020; Yang Y. et al., 2020). In consonance with the above studies, hsa_circ_0001479 overexpression was demonstrated to promote GC cell proliferation and migration by functioning as a sponge for miR-133a-5p, which resulted in the upregulation of DEK expression (Zang et al., 2023).

Pearson’s correlation analysis indicated an inverse relationship in the expression of miR-133a and circ-104792 in the chorion and decidua tissues of patients with RSA. By utilizing the TargetScan website, a possible binding site was predicted between circ-104792 and miR-133a. Our results indicate that both miR-133a and circ-104792 are involved in regulating Bcl-xL levels in HTR-8/SVneo and HESC cells *in vitro*. It is well-established that p53 interacts with Bcl-xL and BCL-2, leading to BAX release from Bcl-xL when p53 binds to it (Mihara et al., 2003; Chipuk et al., 2004). Patrick J. Rochette and colleagues observed that UV stimulation of young fibroblasts resulted in increased BAX protein levels and decreased Bcl-xL protein levels, enhancing the BAX/Bcl-xL ratio and promoting cell apoptosis (Rochette and Brash, 2008). Therefore, Bcl-xL is crucial to regulate intrinsic apoptosis, mitochondrial bioenergetics and oxidative stress, even overexpression of Bcl-xL protected cells against the apoptosis and participated in cellular processes (Mas-Bargues et al., 2021; Per et al., 2023). In our study, it was found that the level of Bcl-xL was downregulated in miR-133a transfection group and cell apoptosis was increased in HTR-8/SVneo and HESCs. In addition, the level of Bcl-xL was increased after miR-133a inhibiting or circ-104792 overexpression and cell proliferation was increased compared to miR-133a transfection group both in HTR-8/SVneo and HESCs. These results indicated that miR-133a and circ-104792 could regulated the growth of HTR-8/SVneo and HESCs in the opposite manner.

Moreover, the results from both the luciferase reporter and RNA pull-down assays indicated that miR-133a suppressed the proliferation of HTR-8/SVneo cells while enhancing apoptosis, while circ-104792 counteracted these effects by modulating Bcl-xL expression. IGFBP-1 and PRL are key markers of decidualization in HESCs, and their reduced expression is associated with decreased cell proliferation and negatively correlates with RSA (Yang et al., 2022; Zhou et al., 2022). In our study, miR-133a overexpression led to a decrease in IGFBP-1 and PRL levels, whereas overexpression of circ-104792 resulted in increased levels of these markers in HESCs. Additionally, co-transfection with both miR-133a and circ-104792 mitigated the reduction in IGFBP-1 and PRL caused by miR-133a.

Therefore, the influence of miR-133a and circ-104792 through the regulation of Bcl-xL in RSA represents a significant finding that merits further investigation within a broader pathophysiological context.

Our findings on the miR-133a/circ-104792/Bcl-xL axis, which primarily regulates cell survival and decidualization, may intersect with well-established immune and hormonal mechanisms in RSA. For instance, the maternal-fetal interface is a highly immune-regulated environment. It is plausible that the apoptosis of trophoblasts and dysfunctional decidual cells resulting from miR-133a overexpression could lead to the release of damage-associated molecular patterns (DAMPs), subsequently triggering inappropriate local inflammatory responses (Tang and Hu, 2024). Furthermore, as decidualization is profoundly influenced by hormonal signaling, particularly progesterone, future studies should investigate whether this axis is modulated by hormonal cues, thereby linking endocrine dysfunction to cellular apoptosis at the implantation site.

A key question arising from our study is the potential of circ-104792 as a non-invasive biomarker. CircRNAs are known to be enriched and stable in exosomes, which are actively secreted into bodily fluids including maternal blood (Zhang et al., 2023). While our study confirmed the downregulation of circ-104792 in placental tissues, its presence and diagnostic value in circulating exosomes from RSA patients remain to be explored. We propose that measuring exosomal circ-104792 levels in maternal serum could represent a promising strategy for the early identification of RSA, a hypothesis that warrants validation in future clinical cohorts.

From a therapeutic perspective, the circ-104792/miR-133a axis presents two attractive nodal points for intervention. To inhibit the pathogenic miR-133a, antisense oligonucleotides (ASOs) or antagomirs could be developed to specifically silence its function and restore Bcl-xL expression. Conversely, strategies to elevate the protective circ-104792, such as the use of plasmid or virus-based vectors for its overexpression, could effectively ‘sponge’ endogenous miR-133a. Although the delivery of such nucleic acid-based therapeutics to the maternal-fetal interface poses significant challenges, recent advances in nanoparticle-based targeted delivery systems offer a potential pathway for future application (Qu et al., 2024). In conclusion, our findings revealed that miR-133a attenuates via Bcl-xL in RSA, and circ-104792 promoted cell proliferation and dysfunction via Bcl-xL in the opposite manner. And circ-104792 binding to miR-133a and it play an important role in cell proliferation and dysfunction of trophoblast and decidualization of HESCs in RSA. Our study, primarily conducted *in vitro*, identifies the circ-104792/miR-133a/Bcl-xL axis as a potential regulatory network in RSA. However, the underlying mechanisms require further validation, particularly *in vivo*. Our study provides initial evidence for the functional significance of the circ-104792/miR-133a/Bcl-xL axis in regulating trophoblast and decidual stromal cell behaviors associated with RSA. It is important to note that these findings are primarily derived from *in vitro* models, and their pathophysiological relevance *in vivo* awaits further investigation. Future studies with larger clinical cohorts and animal models are essential to validate the potential of this axis as a diagnostic biomarker or therapeutic target.

## Data Availability

The original contributions presented in the study are included in the article/Supplementary Material, further inquiries can be directed to the corresponding author.
