# Supplementary material for: Circ-104792/miR-133a/Bcl-xL influences the proliferation and function of human trophoblastic and decidual stromal cells involved in recurrent abortion disease
Source: Front Genet. 2026 Feb 12;17:1707900. doi: 10.3389/fgene.2026.1707900 (PMC12935320; doi:10.3389/fgene.2026.1707900)
Supplement: Supplementary file 2 [file Table1.docx]

Table S1: primer sequences for qRT-PCR

| **Gene** | **Primer** | **Sequence (5'-3')** |
| --- | --- | --- |
| β-actin | Forward | CCCTGGAGAAGAGCTACGAG |
|  | Reverse | CGTACAGGTCTTTGCGGATG |
| U6 | Forward | CGCTTCGGCAGCACATATAC |
|  | Reverse | AAATATGGAACGCTTCACGA |
| miR-133a | loop primer | GTCGTATCCAGTGCAGGGTCCGAGGTATTCGCACTGGATACGACAGCCTATC |
|  | Forward | TGCGCTTTGGTCCCCTTCAACC |
|  | Reverse | CCAGTGCAGGGTCCGAGGTATT |
| circ-104792 | loop primer | GTCGTATCCAGTGCAGGGTCCGAGGTATTCGCACTGGATACGACCAGCTG |
|  | Forward | AAGAAGTGGCTGTAGGGAGCATAG |
|  | Reverse | TGCCTCACAGAACAGTCTCCATAC |
| BCL-XL | Forward | CTGAATCGGAGATGGAGACC |
|  | Reverse | TGGGATGTCAGGTCACTGAA |
