# Supplementary material for: Circ-104792/miR-133a/Bcl-xL influences the proliferation and function of human trophoblastic and decidual stromal cells involved in recurrent abortion disease
Source: Front Genet. 2026 Feb 12;17:1707900. doi: 10.3389/fgene.2026.1707900 (PMC12935320; doi:10.3389/fgene.2026.1707900)
Supplement: Supplementary file 4 [file DataSheet1.pdf]

Uncropped Films Exposed to western blot membranes in this study

Fig 2F, upper panel:

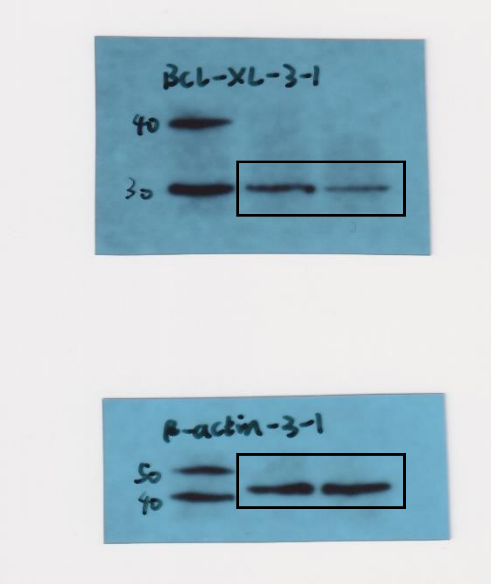

Fig 2F, button panel:

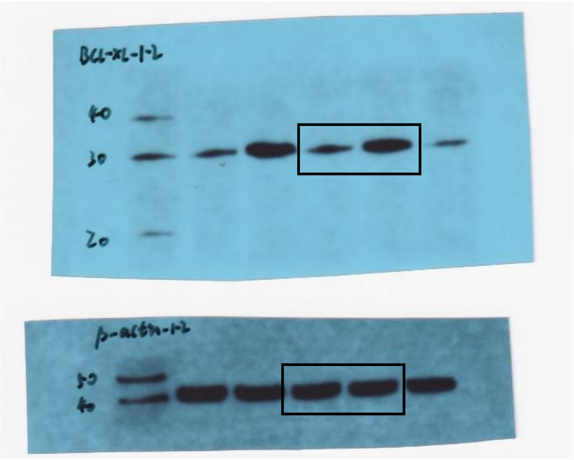

Fig 3F, upper panel:

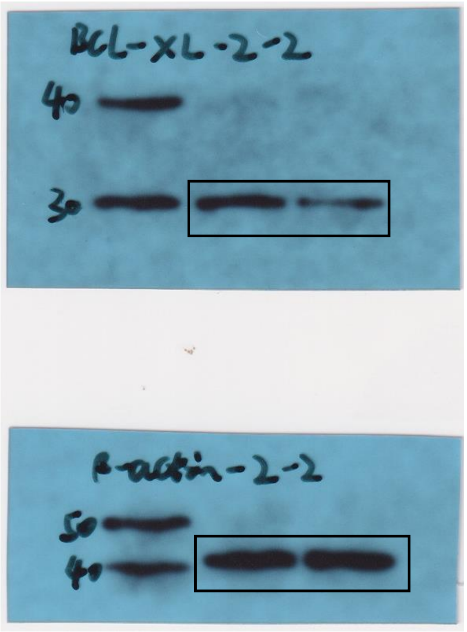

Fig 3F, button panel:

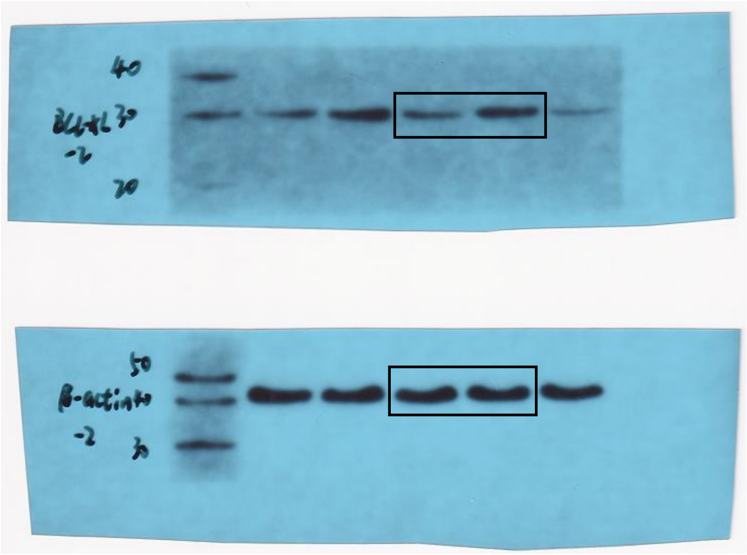

Fig 4H:

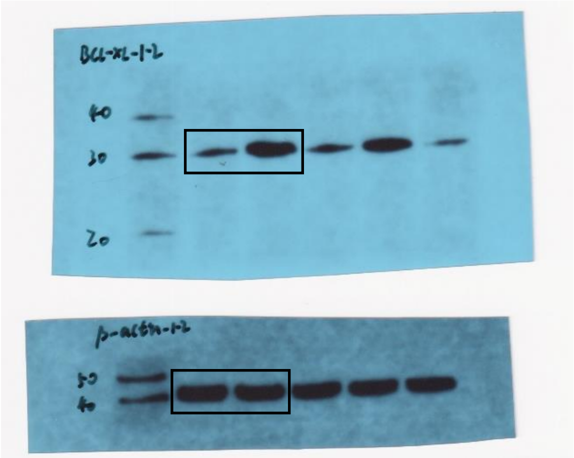

Fig 5F:

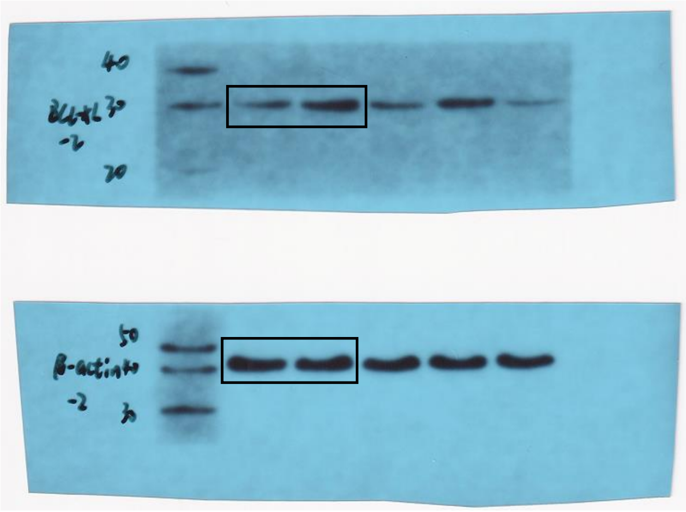

Fig 7H:

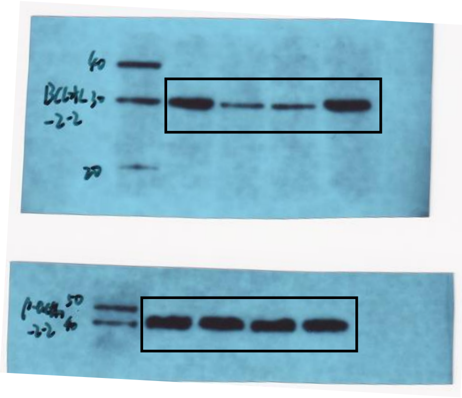
40
BCL-XL 30
-2-2
20
β-actin 50
-2-2 40

Fig 8G:

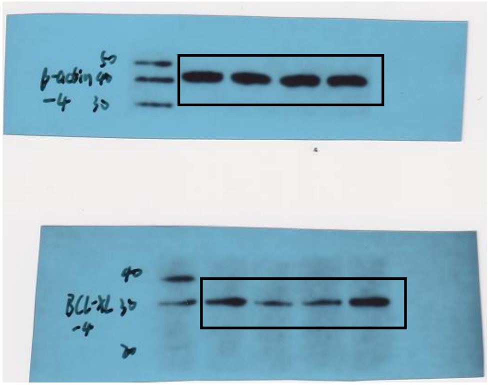
50
β-actin 40
-4 30
40
BCL-XL 30
-4
20
